# Atrial fibrillation in patients with first-ever stroke: Incidence trends and antithrombotic therapy before the event

**DOI:** 10.1371/journal.pone.0209198

**Published:** 2018-12-19

**Authors:** Yo Han Jung, Young Dae Kim, Jinkwon Kim, Sang Won Han, Kyung-Yul Lee

**Affiliations:** 1 Department of Neurology, Changwon Fatima Hospital, Changwon, Gyeongsangnam-do, Korea; 2 Department of Neurology, Yonsei University College of Medicine, Seoul, Korea; 3 Department of Neurology, CHA Bundang Medical Center, CHA University, Seongnam, Gyeonggi-do, Korea; 4 Department of Neurology, Inje University College of Medicine, Seoul, Korea; 5 Severance Institute for Vascular and Metabolic Research, Yonsei University College of Medicine, Seoul, Korea; University of Miami School of Medicine, UNITED STATES

## Abstract

**Background:**

Atrial fibrillation (AF) is the most common cardiac arrhythmia among adults. Despite the proven advantages in primary and secondary stroke prevention in patients with AF, oral anticoagulation (OAC) therapy is still underused in many countries. In this study, we investigated the incidence of AF-related ischemic stroke over the past decade in South Korea and trends of preventive antithrombotic therapy use before stroke in a nationwide cohort.

**Methods and findings:**

The data source for this study was a nationwide sample cohort comprising 1,025,340 individuals that was established by the nationwide health insurance system in 2002. A total of 10,215 patients with acute ischemic stroke (AIS) were selected from the cohort between 2004 and 2013. AF was identified in 1,662 patients, and 979 patients had preexisting AF before AIS. The annual proportion of patients with AIS with AF gradually increased from 13.4% to 22.6% over the study period (p for trends <0.001). Only 14.0% of patients with high risk AF were receiving OAC before the stroke, and this proportion remained relatively constant during the study period. However, the proportion of patients treated with antiplatelet agents had increased from 18.8% in 2004 to 45.3% in 2013, while that of patients receiving no antithrombotic agent decreased from 64.6% in 2004 to 43.9% in 2013. As a limitation, no information was available about non-vitamin K antagonist oral anticoagulants, because they were widely used since late 2014 in Korea.

**Conclusions:**

The number of patients with AIS and AF has steadily increased over the last 10 years in Korea. However, a small portion of patients with AF were receiving OAC therapy before the stroke and about half of the patients did not receive any antithrombotic medication. Our study demonstrates that there is huge gap between clinical practice and treatment guidelines for patients with AF in Korea.

## Introduction

Atrial fibrillation (AF) is the most common cardiac arrhythmia among adults[1]. The presence of AF independently increases the risk of stroke and stroke-related death, resulting in high health-care costs and public health burden [2–4]. The risk of stroke in AF can be effectively reduced with oral anticoagulant (OAC) therapy [2]. However, despite its proven advantage and guideline recommendations for primary and secondary stroke prevention in AF, antithrombotic therapy has been reported to be still underused in many countries [5–7].

Globally, the prevalence of AF has increased [8,9]. The number of patients with AF is expected to increase 2.5-fold during the next 50 years, along with the growing elderly population [8]. Similar increasing trends of AF are reported for South Korea [10,11]. However, there has been very little data on the incidence trend of AF-related stroke and patterns of optimum antithrombotic therapy before the stroke over the past decade in South Korea.

In this study, we investigated whether there were changes in the incidence trend of AF-related stroke in South Korea between 2004 and 2013 as well as in the preventive antithrombotic therapy pattern before hospitalization for ischemic stroke, using data from a nationwide sample cohort.

## Methods

### NHIS sample cohort

The data source for this study was the Korean Nationwide Health Insurance System (NHIS). The NHIS is a mandatory universal health insurance system that covers most of the South Korean population (~97%). It includes a centralized healthcare claims database that provides a nationwide source of information on healthcare resource utilization [10]. The Health Insurance Review Agency (HIRA), which reviews medical billing and claims (including diagnostic information) from all providers, ensures quality control for this system [12].

In 2002, the NHIS established a nationwide sample cohort comprising 1,025,340 individuals (2% of the entire population of South Korea) [13,14]. The data were extracted with stratified and systematic sampling methods, considering the age and sex of all patients who used medical services [15]. The cohort composition was adjusted by age, sex, and income level, so that the entire population is represented regarding both demographic factors as well as their socioeconomic status. This cohort had a semi-dynamic design. When an individual was dropped due to death or emigration, a newborn baby was newly recruited to maintain the cohort size. From the cohort database, we used claims data such as diagnostic codes, presence of hospitalization, codes for medical examination including brain imaging, dates of medical resource use, and prescribed drugs for all individuals [16].

### Selection of patients

We included only patients with first-ever ischemic stroke from the sample cohort for this analysis. The inclusion criteria were as follows: (1) patients who were hospitalized with acute ischemic stroke (AIS), indicated by the code “I63” in the Korean Classification of Disease-6 (KCD-6) system which is comparable to the International Classification of Disease-10 (ICD-10) system, as primary condition from January 1, 2004 to December 31, 2013; (2) patients who underwent brain computed tomography or magnetic resonance imaging. By these criteria we included patients with AIS who presumably had confirmed their ischemic stroke with brain imaging. The exclusion criteria were as follows: (1) patients with previous ischemic stroke before 2004, excluding patients with code I63 between 2002 and 2003 to avoid cases with pre-existing ischemic stroke as incident cases; (2) patients with duplicated data because of a transfer to another hospital within one day after stroke onset.

### Clinical variables

We collected data on comorbidities for each patient using diagnostic codes based on the KCD-6. We identified the presence of clinical variables on the basis of any inpatient or outpatient claim data during the study period. Using the KCD-6 code, we determined whether patients had AF (I48.0), hypertension (I10), diabetes mellitus (DM) (E10-E14), ischemic heart disease (I20-I25), congestive heart failure (I11.0, I13.0, I13.2, I50.0, I50.1, I50.9), chronic kidney disease (CKD) (N18-N19), transient ischemic attack (TIA) (G450), and vascular diseases (I70 for atherosclerosis of aorta, I74 for systemic embolism [arterial embolism and thrombosis], I20-I23 for acute myocardial infarction) [17]. When these comorbidities were detected within 90 days of admission, we concluded that the patient had comorbidities.

### CHA_2_DS_2_-VASc score & ATRIA score

We calculated the CHA_2_DS_2_-VASc score for each patient based on previous published criteria [18]. Briefly, we assigned 2 points for age 75 years or older and previous stroke/TIA/systemic thromboembolism, and 1 point for congestive heart failure, hypertension, DM, vascular disease, and age 65 to 74 years. The high risk group was defined as having a CHA_2_DS_2_-VASc score ≥ 2.

We also calculated the ATRIA bleeding score which has been proposed for stratifying the bleeding risk for patients on warfarin. The score was calculated in each patient on the basis of the method previously described [8] However, for determining the history of anemia or severe renal disease, we used KCD-6 codes because the results of laboratory tests were not available in the NHIS database. Using KCD-6 codes, we identified anemia (D46, D50-53, D55-61, D63-64), severe renal disease (CKD, stage 4–5 [N184, N185]), age ≥75 years, and prior hemorrhage (I60- 62 for intracranial hemorrhage, K25.0, K25.2, K25.4, K25.6 for gastric ulcer with hemorrhage, K92.2 for gastrointestinal hemorrhage) [15]. For the calculation of the ATRIA bleeding score, 3 points were assigned for anemia or severe renal disease, 2 points for age ≥ 75, and 1 point for prior hemorrhage or hypertension.

### Pre-stroke antithrombotic therapy

We identified medication prior to hospitalization for AIS treatment. For this, we extracted the codes for antithrombotic agents by using the Anatomical Therapeutic Chemical classification (“B01AC” for antiplatelet agents and “B01A” for OAC) and the Korean ingredient codes for distinguishing the specific drugs [15]. We also checked the prescription dates. Patients on antithrombotic therapy were considered as managed when they were receiving a regimen including warfarin or antiplatelet agents (aspirin, clopidogrel, ticlopidine, cilostazol, or triflusal) within 120 days before their admission for an AIS event [19]. When an individual had not received any antithrombotic regimen within 120 days before admission, they were classified into the “no treatment” group. We divided the data, based on treatment patterns, into three groups, namely a “no treatment” group, an “only antiplatelet agents” group, and an “OAC” group with/without antiplatelet agents.

### Statistical analysis

Continuous variables are presented as mean ± standard deviation, whereas categorical variables are expressed as numbers (percentages). We categorized the data by age, into bins of <40, 40–49, 50–59, 60–69, 70–79, and ≥80 years. Trends in AF incidence over time were investigated using a logistic regression model with AF as the dependent variable. We also used logistic regression models adjusted for all variables including age, sex, hypertension, DM, congestive heart failure, hyperlipidemia, ischemic heart disease, CKD, peripheral arterial disease, previous TIA, vascular diseases, hemorrhage, and the presence of anemia to identify the independent contributing factors for the use of OAC therapy. Potential determinants based on the results of univariate analysis (p<0.1) and variables which were already known to be related to OAC therapy were selected for entry into the multivariate analysis. Two-sided p-values of 0.05 were considered statistically significant. All analyses were performed using SAS statistical software, version 9.4 (SAS Institute Inc., Cary, NC, USA).

This study was approved by the institutional review boards of the HIRA.

## Results

**Fig 1:**
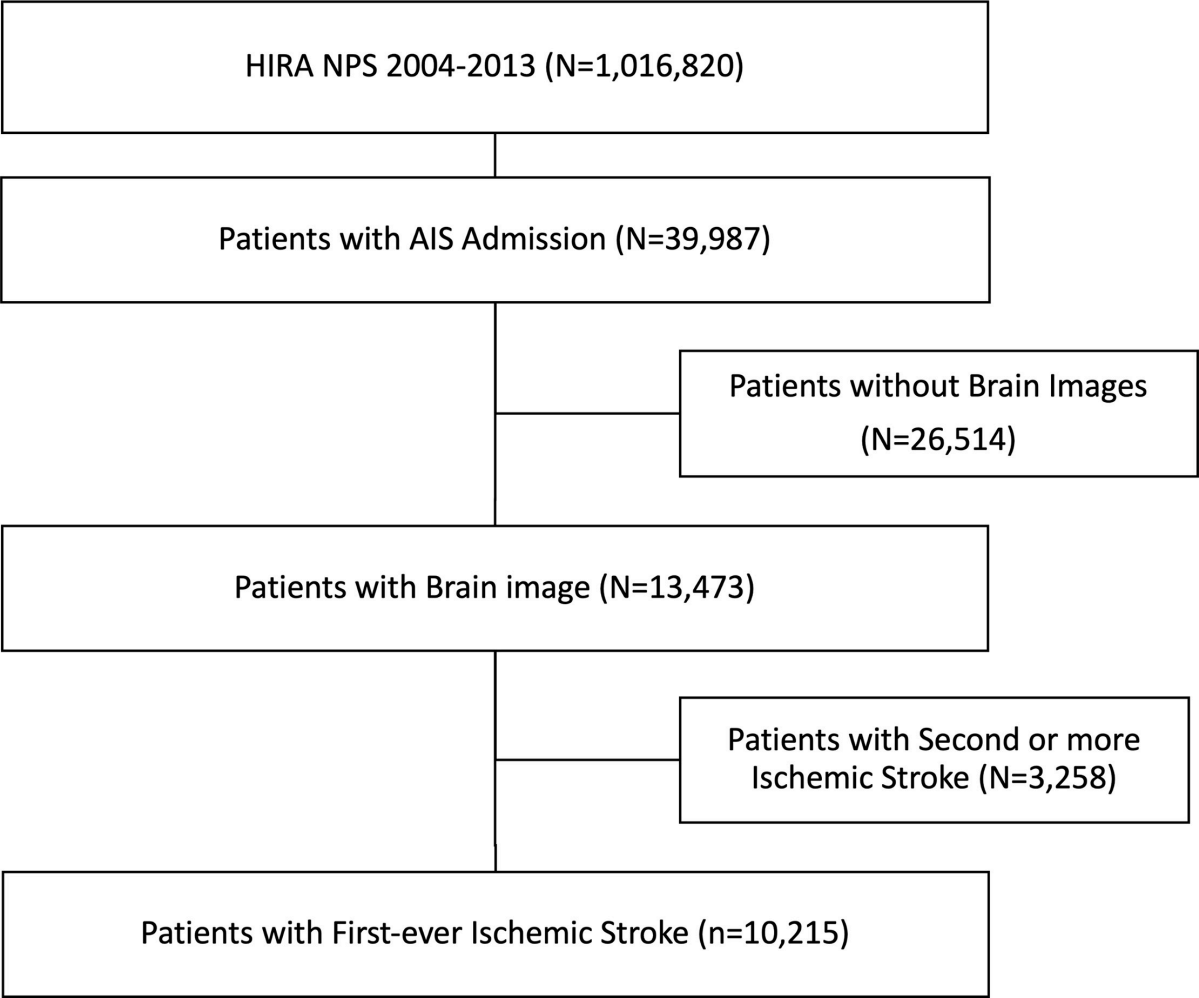
Flow chart showing inclusion and exclusion criteria, the Nationwide Health Insurance System sample cohort of Korea, 2004–2013.

From the nationwide sample cohort comprising 1,016,820 individuals, we selected subjects who had experienced AIS (n = 39,987) between January 2004 and December 2013. We excluded patients without brain imaging data (n = 26,514) and patients with a previous history of ischemic stroke (n = 3,258). Finally, 10,215 patients with first-ever AIS were selected for this analysis (Fig 1).

Baseline characteristics of the patients with AIS are presented in Table 1. The proportion of patients older than 70 years was 64.0%, and 5,449 (53.3%) patients were male. The most common comorbidity was hypertension (63.7%), followed by hyperlipidemia (49.0%), ischemic heart disease (30.5%), and DM (28.6%). AF was identified in 1,662 (16.3%) patients with AIS, and AF was diagnosed before AIS in 979 (58.9%) patients. Compared to patients with AIS without AF, those with AF were more likely to be older or female and had a higher prevalence of hypertension, hyperlipidemia, congestive heart failure, ischemic heart disease, CKD, peripheral arterial disease, TIA, vascular disease, anemia, and severe renal disease. However, DM was more common in patients without AF (all p<0.05) (Table 1).

**Table 1:** Baseline characteristics of study population.

Variables	AF (N = 1,662, 16.3%)	No AF (N = 8,553, 83.7%)	All Stroke (N = 10,215)	p value
**Preadmission Factors**				
**Age Group**				< 0.001
**<40**	10 (0.6)	111 (1.3)	121 (1.2)	
**40–49**	27 (1.6)	346 (4.1)	373 (3.7)	
**50–59**	88 (5.3)	1,005 (11.8)	1,093 (10.7)	
**60–69**	271 (16.3)	1,821 (21.3)	2,092 (20.5)	
**70–79**	502 (30.2)	2,545 (29.8)	3,047 (29.8)	
**> = 80**	764 (46.0)	2,725 (31.9)	3,489 (34.2)	
**Female**	825 (49.6)	3,941 (46.1)	4,766 (46.7)	0.008
**Hypertension**	1,258 (75.7)	5,251 (61.4)	6,509 (63.7)	< 0.001
**Diabetes mellitus**	431 (25.9)	2,493 (29.2)	2,924 (28.6)	0.008
**Congestive Heart Failure**	701 (42.2)	954 (11.2)	1,655 (16.2)	< 0.001
**Hyperlipidemia**	999 (60.1)	4,008 (46.9)	5,007 (49.0)	< 0.001
**Ischemic Heart Disease**	869 (52.3)	2,248 (26.3)	3,117 (30.5)	< 0.001
**Chronic Kidney Disease**	144 (8.7)	496 (5.8)	640 (6.3)	< 0.001
**Peripheral Arterial Disease**	178 (10.7)	382 (4.5)	560 (5.5)	< 0.001
**TIA**	379 (22.8)	1,541 (18.0)	1,920 (18.8)	< 0.001
**Vascular Disease**	801 (48.2)	1,969 (23.0)	2,770 (27.1)	< 0.001
**Anemia**	648 (39.0)	2,567 (30.0)	3,215 (31.5)	< 0.001
**Severe Renal Disease**	11 (0.7)	39 (0.5)	50 (0.5)	< 0.001
**Prior Hemorrhage**	336 (20.2)	1,038 (12.1)	1,374 (13.5)	0.271
**CHA**_**2**_**DS**_**2**_**-VASc Score**				< 0.001
**0**	36 (2.2)	492 (5.8)	528 (5.2)	
**1**	55 (3.3)	947 (11.1)	1,002 (9.8)	
**2**	153 (9.2)	1,410 (16.5)	1,563 (15.3)	
**3**	255 (15.3)	1,730 (20.2)	1,985 (19.4)	
**4**	315 (19)	1,636 (19.1)	1,951 (19.1)	
**5**	355 (21.4)	1,191 (13.9)	1,546 (15.1)	
**6**	267 (16.1)	713 (8.3)	980 (9.6)	
**7**	151 (9.1)	309 (3.6)	460 (4.5)	
**8**	63 (3.8)	103 (1.2)	166 (1.6)	
**9**	12 (0.7)	22 (0.3)	34 (0.3)	
**ATRIA Score**				< 0.001
**0**	156 (9.4)	1,959 (22.9)	2,115 (20.7)	
**1**	282 (17.0)	1,675 (19.6)	1,957 (19.2)	
**2**	186 (11.2)	1,172 (13.7)	1,358 (13.3)	
**3**	532 (32.0)	2,023 (23.7)	2,555 (25.0)	
**4**	134 (8.1)	583 (6.8)	717 (7.0)	
**5**	76 (4.6)	311 (3.6)	387 (3.8)	
**6**	241 (14.5)	693 (8.1)	934 (9.1)	
**7**	51 (3.1)	117 (1.4)	168 (1.6)	
**8**	0 (0.0)	3 (0.0)	3 (0.0)	
**9**	3 (0.2)	12 (0.1)	15 (0.1)	
**10**	1 (0.1)	5 (0.1)	6 (0.1)	

AF, atrial fibrillation; TIA, transient ischemic attack

### Temporal changes in incidences of AIS with AF

**Fig 2:**
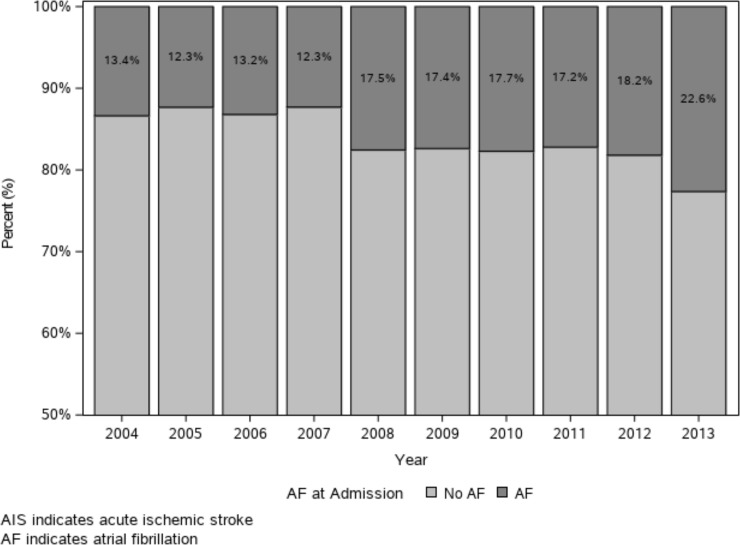
Proportion of acute ischemic stroke with atrial fibrillation from 2004 to 2013.

The temporal incidences of AIS with or without AF are shown in Table 2. Although total numbers of patients with AIS are fluctuating, the proportion of patients with AIS with AF gradually increased from 2004 (13.4%) to 2013 (22.6%) (p for trends <0.001), indicating a relative increase of 68.7% over the course of 10 years (Fig 2).

**Table 2:** Number of AIS patients with AF by year.

Year	2004	2005	2006	2007	2008	2009	2010	2011	2012	2013	Total
**No AF**	648 (86.6)	975 (87.7)	894 (86.8)	934 (87.7)	874 (82.5)	761 (82.6)	781 (82.3)	948 (82.8)	877 (81.8)	861 (77.4)	8,553 (83.7)
**AF**	100 (13.4)	137 (12.3)	136 (13.2)	131 (12.3)	186 (17.6)	160 (17.4)	168 (17.7)	197 (17.2)	195 (18.2)	252 (22.6)	1,662 (16.3)
**Known**	51 (51.0)	71 (51.8)	72 (52.9)	75 (57.3)	114 (61.3)	89 (55.6)	108 (64.3)	116 (58.9)	132 (67.7)	151 (59.9)	979 (58.9)
**New**	49 (49.0)	66 (48.2)	64 (47.1)	56 (42.7)	72 (38.7)	71 (44.4)	60 (35.7)	81 (41.1)	63 (32.3)	101 (40.1)	683 (41.1)
**Total**	748	1,112	1,030	1,065	1,060	921	949	1,145	1,072	1,113	1,0215

p for trends <0.001

Values are represented as numbers (%).

AIS, acute ischemic stroke; AF, atrial fibrillation

In terms of risk levels based on CHA_2_DS_2_-VASc scores, the proportion of patients falling into the high-risk group (CHA_2_DS_2_-VASc score of ≥ 2 before AIS) increased annually (S1 Table).

### Antithrombotic medication pattern before AIS

**Fig 3:**
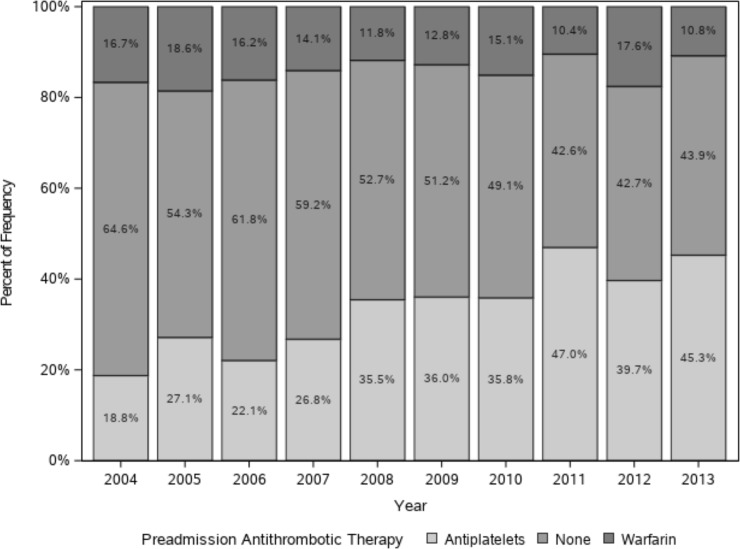
Preadmission antithrombotic therapy in OAC therapy candidates*. * Patients with CHA_2_DS_2_-VASc score ≥2 before acute ischemic stroke event were selected.

**Fig 4:**
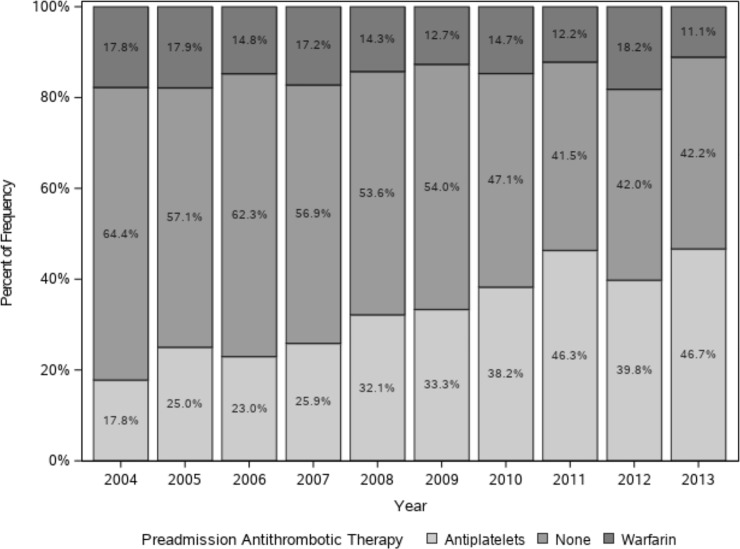
Preadmission antithrombotics therapy in OAC therapy candidates without high bleeding risk*. * CHA_2_DS_2_-VASc score ≥ 2 and a lower bleeding risk (ATRIA score ≤ 4) before acute ischemic stroke event were calculated.

Among 979 patients with AIS with preexisting AF, 134 patients (13.7%) had been on OAC therapy, while 347 patients (35.4%) had been treated with antiplatelet agents. Among the antiplatelet agents, aspirin was the most common regimen (204, 58.8%), followed by clopidogrel (93, 26.8%). A total of 498 patients (50.9%) did not receive any antithrombotic therapy before AIS (S2 Table). When we analyzed 953 patients with a high risk for thromboembolism (CHA_2_DS_2_-VASc score ≥ 2), 133 patients (14.0%) had been on OAC therapy, and 343 patients (36.0%) on antiplatelet agents, while 477 patients (50.1%) had not received any antithrombotic therapy (Table 3). The proportion of patients receiving OAC therapy fluctuated during the study period between 10.4% and 18.6%. However, the proportion of patients taking antiplatelet agents had increased from 18.8% in 2004 to 45.3% in 2013, while that of patients in the no treatment group decreased from 64.6% in 2004 to 43.9% in 2013 (p = 0.015) (Fig 3). To investigate the proportion of patients with AF receiving OAC therapy without a high potential for bleeding complications, we selected patients with a CHA_2_DS_2_-VASc score ≥ 2 and an ATRIA score ≤ 4 as OAC therapy candidates(Fig 4). Among these 695 patients, only 14.8% (103/695) had been treated with OAC therapy before the stroke.

**Table 3:** Preadmission antithrombotic therapy in OAC therapy candidates*.

Treatment	Year										
	2004	2005	2006	2007	2008	2009	2010	2011	2012	2013	Total
**Antiplatelets**	9 (18.8)	19 (27.1)	15 (22.1)	19 (26.8)	39 (35.5)	31 (36.0)	38 (35.8)	54 (47.0)	52 (39.7)	67 (45.3)	343 (36.0)
**None**	31 (64.6)	38 (54.3)	42 (61.8)	42 (59.2)	58 (52.7)	44 (51.2)	52 (49.1)	49 (42.6)	56 (42.7)	65 (43.9)	477 (50.1)
**Warfarin**	8 (16.7)	13 (18.6)	11 (16.2)	10 (14.1)	13 (11.8)	11 (12.8)	16 (15.1)	12 (10.4)	23 (17.6)	16 (10.8)	133 (14.0)
**Total**	48	70	68	71	110	86	106	115	131	148	953

p for trends = 0.015

* Patients with CHA_2_DS_2_-VASc score ≥2 before acute ischemic stroke event were selected.

Values are represented as numbers (%).

**Fig 5:**
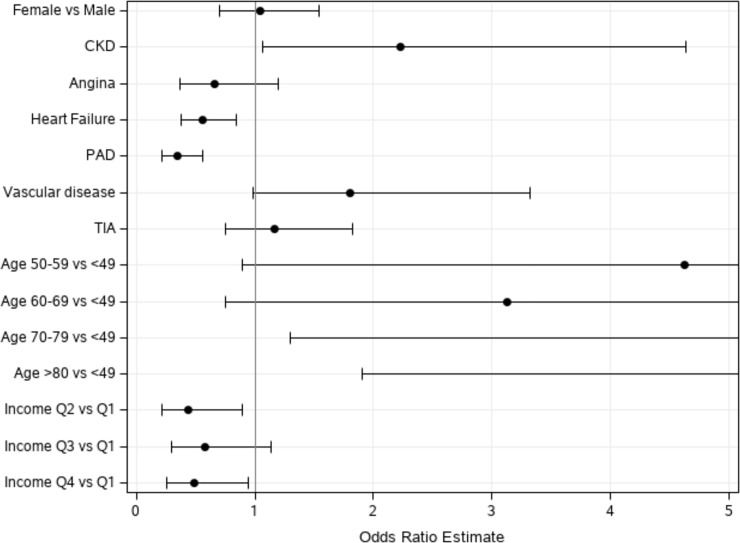
Multivariate regression analysis for underuse of OAC in patients with atrial fibrillation*. * CHA_2_DS_2_-VASc score ≥ 2 before acute ischemic stroke events were taken into account.

To identify independent factors for OAC non-use in AIS with AF and a CHA_2_DS_2_-VASc score ≥ 2, we performed univariate and multiple logistic analyses (Fig 5). Multivariate logistic regression analysis adjusting for variables such as age, sex, income, congestive heart failure, ischemic heart disease, CKD, peripheral arterial disease, TIA, and vascular disease revealed that patients were like to receive OAC therapy when they had a history of peripheral arterial disease (OR 0.34, 95% CI 0.21–0.56) and congestive heart failure (OR 0.56, 95% CI 0.37–0.84), while OAC was not used in patients with CKD (OR 2.23, 95% CI 1.07–4.64). Compared to patients at the age of 50 years or younger, the odds of receiving no OAC therapy were significantly higher in those aged 70–79 years (OR 5.3, 95% CI 1.29–21.67) and in those aged 80 years or older (OR 7.79, 95% CI 1.9–31.88) (Table 4). A similar trend was found when we analyzed a subgroup of patients with a CHA_2_DS_2_-VASc score ≥ 2 and an ATRIA score ≤ 4 (S3 Table and S1 Fig).

**Table 4:** Determinants for OAC non-use among patients with OAC candidates*.

	Unadjusted Model				Multivariate Model			
	OR	95% CI		p-value	OR	95% CI		p-value
**Age, year**								
50~59 vs ~49	3.87	0.8	18.81	0.004	4.63	0.89	24.07	0.069
60~69 vs ~49	2.94	0.75	11.62		3.13	0.75	13.1	0.118
70~79 vs ~49	5.09	1.31	19.72		5.3	1.29	21.67	0.02
80~ vs ~49	6.48	1.69	24.94		7.79	1.9	31.88	0.004
**Gender**								
Female vs Male	1.1	0.77	1.59	0.597	1.04	0.7	1.54	0.847
**Income**								
Low to Moderate vs Low	0.43	0.22	0.85	0.108	0.44	0.22	0.89	0.023
Moderate to High vs Low	0.55	0.29	1.08		0.58	0.29	1.14	0.113
High vs Low	0.51	0.27	0.96		0.49	0.25	0.94	0.033
**Hypertension**	0.98	0.59	1.64	0.951				
**Diabetes mellitus**	0.95	0.63	1.43	0.811				
**Congestive Heart Failure**	0.62	0.42	0.91	0.015	0.56	0.37	0.84	0.005
**Hyperlipidemia**	1.19	0.8	1.76	0.388				
**Ischemic Heart Disease**	0.82	0.55	1.24	0.35	0.66	0.37	1.19	0.171
**Chronic Kidney Disease**	1.85	0.91	3.76	0.09	2.23	1.07	4.64	0.033
**Peripheral Arterial Disease**	0.41	0.26	0.63	<0.001	0.34	0.21	0.56	<0.001
**TIA**	1.19	0.78	1.83	0.416	1.17	0.75	1.82	0.496
**Vascular Disease**	0.98	0.65	1.48	0.934	1.81	0.98	3.32	0.057
**Anemia**	1.19	0.82	1.73	0.354				
**Prior Hemorrhage**	>999.99	<0.01	>999.99	0.985				
**Angina**	1.15	0.78	1.7	0.474				
**CHA**_**2**_**DS**_**2**_**-VASc Score**								
3 vs 2	1.01	0.46	2.22	0.182				
4 vs 2	1.62	0.76	3.48					
5 vs 2	1.61	0.77	3.37					
6 vs 2	2.56	1.15	5.73					
7 vs 2	1.19	0.54	2.63					
8 vs 2	1.36	0.49	3.79					
9 vs 2	2.45	0.29	21.03					
**ATRIA Score**								
1 vs 0	1.09	0.45	2.6	0.332				
2 vs 0	1.56	0.58	4.23					
3 vs 0	2.18	0.93	5.12					
4 vs 0	1.56	0.6	4.07					
5 vs 0	1.79	0.56	5.73					
6 vs 0	2.25	0.9	5.63					
7 vs 0	1.77	0.52	6.01					
9 vs 0	>999.99	<0.01	>999.99					
10 vs 0	>999.99	<0.01	>999.99					

* CHA_2_DS_2_-VASc score ≥2 before acute ischemic stroke event was calculated.

CI, confidence interval; OR, odds ratio; TIA, transient ischemic attack

## Discussion

Korea has been reported to experience an increase in the incidence of AF [10,11]. The incidences of AF in patients aged ≥ 70 in Korea was noted as 2.0% in 2000 and 5.7% between 2005 and 2010. In addition, the proportion of AF-related stroke cases relative to the total number of ischemic stroke cases in Korea has been reported as 17.3% in 2009 [20]. According to a global survey of the frequency of AF-associated stroke between 2013 and 2014, the mean proportion in East Asia and Pacific was 22% [21]. Our results show that the number of patients with AF has increased from 13.4% in 2004 to 22.6% in 2013 among patients with AIS.

Although the increasing trend of AF-related AIS may be partially caused by the more extensive diagnostic workup for hidden embolic sources [22,23], newly detected AF was noted to increase continuously over the years (p for trends < 0.001). Considering that age is one of the strongest risk factors for AF development and that the aging population in Korea is rapidly increasing, the increasing trend of AF-related stroke in Korea could intensify in the future.

Despite the proven efficacy of OAC treatment for stroke prevention, OAC therapy has been used in only 14.4% cases in our AF population. In addition, we have to consider that some of the patients receiving OAC therapy may not be in the therapeutic range for preventing thromboembolism. We do not have information on international normalized ratio (INR) in this cohort data. A recent retrospective analysis of patients with AIS with a known history of AF showed that patients receiving subtherapeutic warfarin were more common than those receiving therapeutic warfarin [24]. Approximately 50% of patients with a high risk for stroke did not receive any antithrombotic therapy. Previous reports showed that 62% to 79% of patients with AF were not receiving adequate OAC therapy in Korea [15,20,25]. The proportion of patients receiving OAC therapy among patients diagnosed with AF is somewhat lower here than in previous studies. A retrospective observational study, which consisted of hospitals participating in the “Get With the Guidelines-Stroke” program reported that 16.4% of patients with AIS with a known history of AF received OAC therapy with warfarin or non-vitamin K antagonist oral anticoagulants (NOACs) [24], and a GLORIA-AF registry phase-1 study reported that 32.8% of patients with AF received OAC therapy [26].

We speculated that there were several reasons for a lower use of OAC in our data. Our study was conducted using data collected before the use of NOACs, which are easier to use than warfarin. GLORIA-AF registry phase-2 study showed that greater portion (79.9%) of subjects with AF received OAC therapy compared to GLORIA-AF registry phase-1 study which included subject before NOACs use [26–28]. In addition, we excluded patients with a previous history of cerebral infarction. Because cerebral infarction is the main indication for OAC treatment in clinical practice, the use of anticoagulants in our study seems to be low. However, we have to consider the difference between prospective registry study and real-world practice cohort study. In registry study, patients had to give informed consent and this process can be a selection bias of higher percentage of participating patients receiving OAC treatment than the proportion seen among all patients.

Although the proportion of patients not receiving OAC was different between studies, due to the different definitions used for antithrombotic use, our results suggest that the physicians’ preference for OAC therapy in high risk patients with AF has not improved in recent years.

However, a steady increase in patients treated with antiplatelet agents was observed. In a previous study, the factor most associated with non-prescription of OAC was the previous use of antiplatelet agents [27]. Antiplatelet therapy does not need regular blood sampling for therapeutic monitoring for some benefit of stroke prevention in the AF population [29]. In addition, the bleeding risk is lower than that of OAC. These facts could lead to physicians preferring the use of antiplatelet agents rather than OAC in patients with AF. However, although antiplatelet agents can prevent ischemic stroke and reduce poor outcome and mortality [29,30], they are not as effective as OAC therapy. Antiplatelet agents are recommended only for selected patients, according to current guidelines. Our results show that there was huge gap between the physicians’ preferences and treatment guidelines.

Our study demonstrates that old age and CKD were associated with the non-use of OAC. This suggests that age and concomitant diseases related to bleeding risk could be strong factors for deciding about antithrombotic therapy in real practice. Physicians seem to be more reluctant to use OAC in older patients with AF because of the potential risk of bleeding caused by fall, renal dysfunction, low cognitive function, or the use of multiple medications [5–7,15,31–34]. Moreover, old age and CKD can be associated with difficulties in maintaining the therapeutic range of warfarin, which could increase the bleeding risk [35]. However, old age is also one of the strongest risk factors for ischemic stroke, and OAC use is beneficial for stroke prevention even in elderly people with AF [36], or those with CKD [37,38].

### Advantages and limitations

A major advantage of our study is the large sample size, using a nationwide sample cohort. Besides, we included patients with AF who had been diagnosed at out-patient clinics as well as those diagnosed at admission, while many previous studies used either out-patient or in-patient cases [11,21], which may underestimate the true incidence of AF.

There are some limitations of this study. First, we did not have access to information including other risk factors such as smoking, physical activity, or body mass index. However, the baseline characteristics of the study population were compatible to those of the Korean Stroke Registry and the proportion (17.4%) of AIS with AF in our study was similar to that of the Stroke Registry (17.3%) [20]. Second, we could not ascertain the type of AF (valvular vs. non-valvular or paroxysmal vs. non-paroxysmal AF). Third, there may be an overestimation of the components of the CHA_2_DS_2_-VASc score or the ATRIA score, due to the inherent limitation of a retrospective cohort study. Lastly, detailed clinical data on INR levels or drug compliance was not available.

NOACs have been approved since 2010 and used in non-valvular AF to prevent ischemic stroke and systemic embolization. Since NOACs were widely used in Korea after insurance coverage on late 2014, we could not get the information about the use of NOACs. Comparable efficacy and lower bleeding complications of NOACs compared to traditional OAC may change the pattern of preventive antithrombotic prescription in patients with AF.

## Conclusions

The numbers of patients with AIS with AF have steadily increased over the last 10 years in Korea. However, only a small portion of patients with AF at high risk for stroke were receiving OAC therapy before they suffered a stroke. Instead, the proportion of patients with AIS who had been treated with antiplatelet agents before their stroke was higher among patients with AF. Our study demonstrates that there is still a gap between clinical practice and optimum treatment for patients with AF in Korea.

## Supporting information

S1 Fig Multivariate regression analysis for underuse of OAC in patients with a high risk for thromboembolism and a lower bleeding risk*. * CHA_2_DS_2_-VASc score ≥ 2 and a lower bleeding risk (ATRIA score ≤ 4) before acute ischemic stroke event were calculated.
(TIFF)

S1 Table Number of patients according to CHA_2_DS_2_-VASc risk classification. (DOCX)

S2 Table Preadmission antithrombotic therapy in patients with known AF before index stroke. (DOCX)

S3 Table Determinants for OAC non-use among patients with a high risk for thromboembolism and a lower bleeding risk. (DOCX)
