# Supplementary figures and images for: Atrial fibrillation in patients with first-ever stroke: Incidence trends and antithrombotic therapy before the event
Source: PLoS One. 2018 Dec 19;13(12):e0209198. doi: 10.1371/journal.pone.0209198 (PMC6300293; doi:10.1371/journal.pone.0209198)

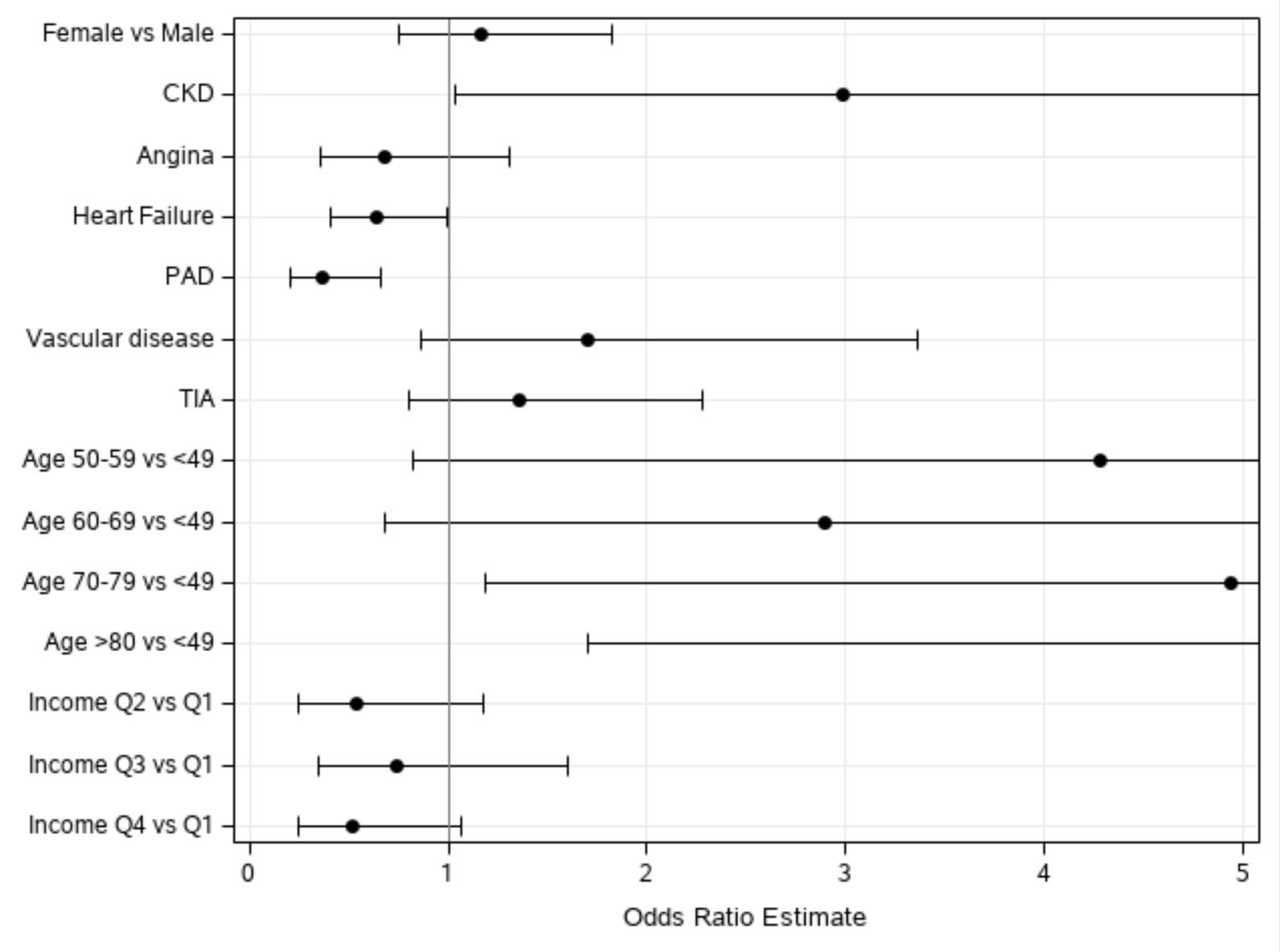

Supplement: S1 Fig — * CHA2DS2-VASc score ≥ 2 and a lower bleeding risk (ATRIA score ≤ 4) before acute ischemic stroke event were calculated. (TIFF) [file pone.0209198.s001.tiff]
