# Supplementary material for: Atrial fibrillation in patients with first-ever stroke: Incidence trends and antithrombotic therapy before the event
Source: PLoS One. 2018 Dec 19;13(12):e0209198. doi: 10.1371/journal.pone.0209198 (PMC6300293; doi:10.1371/journal.pone.0209198)
Supplement: S1 Table — (DOCX) [file pone.0209198.s002.docx]

**S1 Table. Number of patients according to CHA_2_DS_2_-VASc risk classification**

| **CHA_2_DS_2_-VASc risk classification^*^** | **Year** | | | | | | | | | | |
| --- | --- | --- | --- | --- | --- | --- | --- | --- | --- | --- | --- |
|  | **2004** | **2005** | **2006** | **2007** | **2008** | **2009** | **2010** | **2011** | **2012** | **2013** | **Total** |
| **Low**  **(0)** | 5  (5.0) | 2  (1.5) | 7  (5.2) | 4  (3.1) | 8  (4.3) | 4  (2.5) | 6  (3.6) | 5  (2.5) | 3  (1.5) | 10  (4.0) | 54 |
| **Moderate**  **(1)** | 6  (6.0) | 3  (2.2) | 5  (3.7) | 2  (1.5) | 3  (1.6) | 5  (3.1) | 2  (1.2) | 4  (2.0) | 4  (2.1) | 3  (1.2) | 37 |
| **High**  **(≥2)** | 89  (89.0) | 132  (96.4) | 124  (91.2) | 125  (95.4) | 175  (94.1) | 151  (94.4) | 160  (95.2) | 188  (95.4) | 188  (96.4) | 239  (94.8) | 1571 |
| **Total** | 100 | 137 | 136 | 131 | 186 | 160 | 168 | 197 | 195 | 252 | 1662 |

High risk: CHA_2_DS_2_-VASc score ≥ 2; Moderate risk: CHA_2_DS_2_-VASc score =1; Low risk: CHA_2_DS_2_-VASc score =0

Values are represented as numbers (%).
