# Supplementary material for: Atrial fibrillation in patients with first-ever stroke: Incidence trends and antithrombotic therapy before the event
Source: PLoS One. 2018 Dec 19;13(12):e0209198. doi: 10.1371/journal.pone.0209198 (PMC6300293; doi:10.1371/journal.pone.0209198)
Supplement: S2 Table — (DOCX) [file pone.0209198.s003.docx]

**S2 Table. Preadmission antithrombotic therapy in patients with known AF before index stroke**

|  | **2004** | **2005** | **2006** | **2007** | **2008** | **2009** | **2010** | **2011** | **2012** | **2013** | **Total** |
| --- | --- | --- | --- | --- | --- | --- | --- | --- | --- | --- | --- |
| **Warfarin** | 8  (15.7) | 14  (19.7) | 11  (15.3) | 10  (13.3) | 13  (11.4) | 11  (12.4) | 16  (14.8) | 12  (10.3) | 23  (17.4) | 16  (10.6) | 134  (13.7) |
| **Antiplatelets** | 10  (19.61) | 19  (26.8) | 15  (20.8) | 19  (25.3) | 40  (35.1) | 32  (36.0) | 39  (36.1) | 54  (46.6) | 52  (39.4) | 67  (44.4) | 347  (35.4) |
| **None** | 33  (64.7) | 38  (53.5) | 46  (63.9) | 46  (61.3) | 61  (53.5) | 46  (51.7) | 53  (49.1) | 50  (43.1) | 57  (43.2) | 68  (45.0) | 498  (50.9) |
| **Total** | 51 | 71 | 72 | 75 | 114 | 89 | 108 | 116 | 132 | 151 | 979 |

Values are represented as numbers (%).
