# Supplementary material for: Atrial fibrillation in patients with first-ever stroke: Incidence trends and antithrombotic therapy before the event
Source: PLoS One. 2018 Dec 19;13(12):e0209198. doi: 10.1371/journal.pone.0209198 (PMC6300293; doi:10.1371/journal.pone.0209198)
Supplement: S3 Table — (DOCX) [file pone.0209198.s004.docx]

**S3 Table. Determinants for OAC non-use among patients with a high risk for thromboembolism and a lower bleeding risk^*^**

|  | **Unadjusted Model** | | | | **Multivariate Model** | | | |
| --- | --- | --- | --- | --- | --- | --- | --- | --- |
| **Variables** | OR | 95% CI | | p | OR | 95% CI | | p |
| **Age, y** |  |  |  |  |  |  |  |  |
| 50~59 vs ~49 | 3.87 | 0.8 | 18.81 | 0.103 | 4.28 | 0.82 | 22.42 | 0.085 |
| 60~69 vs ~49 | 2.8 | 0.71 | 11.09 | 0.132 | 2.89 | 0.68 | 12.23 | 0.149 |
| 70~79 vs ~49 | 5.02 | 1.28 | 19.66 | 0.016 | 4.94 | 1.19 | 20.55 | 0.028 |
| 80~ vs ~49 | 6.56 | 1.67 | 25.78 | 0.009 | 7.12 | 1.7 | 29.85 | 0.007 |
| **Gender** |  |  |  |  |  |  |  |  |
| Female vs Male | 1.23 | 0.81 | 1.87 | 0.34 | 1.17 | 0.75 | 1.82 | 0.494 |
| **Income** |  |  |  |  |  |  |  |  |
| Low to Moderate vs Low | 0.5 | 0.24 | 1.07 | 0.074 | 0.53 | 0.24 | 1.18 | 0.119 |
| Moderate to High vs Low | 0.71 | 0.34 | 1.5 | 0.372 | 0.74 | 0.35 | 1.6 | 0.448 |
| High vs Low | 0.56 | 0.27 | 1.14 | 0.108 | 0.52 | 0.25 | 1.07 | 0.074 |
| **Hypertension** | 0.94 | 0.53 | 1.67 | 0.829 |  |  |  |  |
| **Diabetes mellitus** | 1.14 | 0.7 | 1.85 | 0.607 |  |  |  |  |
| **Congestive Heart Failure** | 0.69 | 0.45 | 1.06 | 0.093 | 0.64 | 0.41 | 0.99 | 0.048 |
| **Hyperlipidemia** | 1.05 | 0.67 | 1.63 | 0.836 |  |  |  |  |
| **Ischemic Heart Disease** | 0.85 | 0.54 | 1.34 | 0.482 | 0.68 | 0.36 | 1.3 | 0.245 |
| **Chronic Kidney Disease** | 2.64 | 0.94 | 7.43 | 0.067 | 2.99 | 1.04 | 8.61 | 0.043 |
| **Peripheral Arterial Disease** | 0.43 | 0.25 | 0.74 | 0.002 | 0.36 | 0.2 | 0.66 | 0.001 |
| **TIA** | 1.32 | 0.8 | 2.17 | 0.272 | 1.35 | 0.8 | 2.28 | 0.255 |
| **Vascular Disease** | 1.01 | 0.64 | 1.58 | 0.975 | 1.7 | 0.87 | 3.36 | 0.124 |
| **Anemia** | 1.02 | 0.62 | 1.68 | 0.946 |  |  |  |  |
| **Prior Hemorrhage** | 0.89 | 0.54 | 1.48 | 0.662 |  |  |  |  |
| **CHA_2_DS_2_-VASc Score** |  |  |  |  |  |  |  |  |
| 3 vs 2 | 0.91 | 0.41 | 2.02 | 0.821 |  |  |  |  |
| 4 vs 2 | 1.47 | 0.68 | 3.21 | 0.329 |  |  |  |  |
| 5 vs 2 | 1.5 | 0.7 | 3.23 | 0.301 |  |  |  |  |
| 6 vs 2 | 3.35 | 1.32 | 8.47 | 0.011 |  |  |  |  |
| 7 vs 2 | 1.3 | 0.53 | 3.21 | 0.57 |  |  |  |  |
| 8 vs 2 | 1.53 | 0.45 | 5.26 | 0.498 |  |  |  |  |
| 9 vs 2 | >999.99 | <0.01 | >999.99 | 0.988 |  |  |  |  |
| **ATRIA Score** |  |  |  |  |  |  |  |  |
| 1 vs 0 | 1.09 | 0.45 | 2.6 | 0.974 |  |  |  |  |
| 2 vs 0 | 1.56 | 0.58 | 4.23 | 0.566 |  |  |  |  |
| 3 vs 0 | 2.18 | 0.93 | 5.12 | 0.262 |  |  |  |  |
| 4 vs 0 | 1.56 | 0.6 | 4.07 | 0.403 |  |  |  |  |

* CHA_2_DS_2_-VASc score ≥2 and ATRIA score ≤ 4 before acute ischemic stroke event was calculated.

CI, confidence interval; OR, odds ratio; TIA, transient ischemic attack
